# Vibrissal sensing in mammals in a changing world

**DOI:** 10.1242/jeb.250776

**Published:** 2026-02-11

**Authors:** Robyn A. Grant

**Affiliations:** Department of Natural Sciences, Manchester Metropolitan University, Manchester, M1 5GD, UK

**Keywords:** Whiskers, Vibrissa, Somatosensory, Environmental change, Multi-modal

## Abstract

Mammalian vibrissae are part of a specialised, sensitive and precise sensory system. They are involved in multimodal reception, including vibrotactile and electric sensing, which might make them particularly prone to multiple perceptual elements of environmental change. This Review considers the important implications of environmental change for vibrissal form, behaviour and neural signalling. Findings reveal that environmental change can impact all modalities of vibrissal sensing. Increasing exposure to new plant species, pathogens and chemicals in the environment impacts vibrissal growth, sensitivity and neural processing. Acoustic noise and altered air and water flow regimes will cause vibrissal shaft oscillations and may even have the capacity to mask critical stimuli, such as prey signals, although this has not yet been studied. Despite evidence of vibrissal sensing being robust to environmental change in some contexts, such as being able to regulate warm temperatures, many environmental impacts remain poorly understood. There is a need to better understand the levels of pollution and noise in the environment and incorporate these as relevant environmental stimuli in experiments to investigate their impact on vibrissal sensing. Adopting a broader taxonomic focus would also give greater insight into species-specific vibrissal adaptations, many of which can be seen in this Review in response to dietary adaptations. Adopting these approaches in future studies will enhance our understanding of the resilience and vulnerability of vibrissal sensing in the face of rapid environmental change.

## Teaser

**Summary:** The mammalian vibrissal system is precise, sensitive and involved in multimodal sensing. This Review considers the important implications of environmental change for vibrissal sensing.

## Introduction

**Fig. 1.**
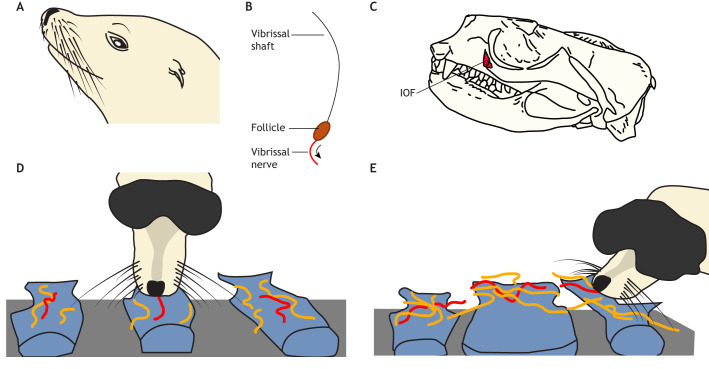
**California sea lion (*Zalophus californianus*) vibrissal morphology and control.** (A) California sea lion vibrissal position and layout. (B) Example vibrissa from a California sea lion. (C) California sea lion skull and highlighted infraorbital foramen (IOF) in red. (D,E) Vibrissal (in orange) and nose (in red) positions of a California sea lion during a texture discrimination task (D) and during a size discrimination task (E) (adapted from Milne et al., 2021).

Facial vibrissae (see Glossary), also called whiskers or tactile hairs, are present across mammals, from marsupials to primates (Fig. 1A,B) (Ahl, 1986; Muchlinski et al., 2020). They are primarily involved in vibrotactile sensing and are mostly studied by neuroscientists to understand more about how the sense of touch is processed in the brain (Campagner et al., 2018; Diamond et al., 2008; Evans et al., 2019; Prescott et al., 2011). Vibrissae themselves are not sensors; rather, they sit within an innervated follicle, which is more sensitive than the follicles of pelage hair (Grant and Goss, 2022). Vibrissae pass bending and vibration information along their shaft to the follicle (Fig. 1B), where it is translated into neural signals for processing throughout the brain, from brainstem (see Glossary) to cortex (Campagner et al., 2018). Mammals are able to apply their vibrissae to guide locomotion around complex habitats in the dark, as well as to hunt, forage and engage in social interactions (Grant and Goss, 2022).

Because vibrissal shafts (see Glossary) pass along vibrotactile information, their shape will affect how they bend and vibrate. Vibrissae of aquatic mammals, such as pinnipeds, tend to be thicker, shorter and more tapered than those of terrestrial mammals, which is thought to make them stiffer and better able to be precisely positioned underwater (Dougill et al., 2020, 2023; Grant et al., 2025). Semi-aquatic species, such as otters and mink, have vibrissae that are somewhat intermediary between terrestrial and aquatic species. Phocid seals even have undulating vibrissae that are oval in cross-section, which is thought to make them streamlined in the water and able to improve the signal-to-noise ratio for hydrodynamic sensing (Hanke et al., 2010). However, it is not just about being aquatic. Nocturnal and arboreal species are more likely to cyclically move their vibrissae to and fro, in a process called whisking (Muchlinski et al., 2020), than aquatic and diurnal, ground-dwelling species. Mammals that whisk have more sensitive vibrissae than those that do not, which has been estimated from the infraorbital foramen size, a hole in the skull that the infraorbital nerve passes through (Fig. 1C) (Muchlinski et al., 2020). The size of the infraorbital foramen is well correlated to the infraorbital nerve diameter and can be used as a proxy for vibrissal sensitivity (Milne et al., 2022; Muchlinski, 2008). Findings suggest that dark, complex, three-dimensional habitats drive important adaptations in vibrissal form and behaviour, especially in animals that climb in the dark and swim underwater (Milne et al., 2022; Muchlinski et al., 2020). Indeed, many of these mammals are often thought to be ‘whisker specialists’ (see Glossary) with highly sensitive and moveable vibrissae (Grant and Arkley, 2016).

Vibrissal movement and behaviour does not just consist of simple whisking movements; rather, it is anticipatory, actively controlled and linked to an animal's attention and perception (Arkley et al., 2014; Grant et al., 2009; Mitchinson and Prescott, 2013). For example, orienting many vibrissae to a salient space in the environment and exploring objects with light sweeping touches are behaviours that we have observed in many mammals, including marsupials, rodents, pinnipeds and eulipotyphls (Grant et al., 2018; Mitchinson et al., 2011; Nakhwa et al., 2024). One of the best examples of vibrissal behaviour can perhaps be seen in the California sea lion (Fig. 1A), which can engage in task-specific movements with their vibrissae (Milne et al., 2021). Specifically, California sea lions move their vibrissae with lateral stroking movements across stimuli to judge textures (Fig. 1D), and around stimuli edges to judge size (Fig. 1E) (Milne et al., 2021) – the same types of behaviours that humans make with their fingertips (Lederman and Klatzky, 1987). It is likely that other species are also capable of such precise and targeted vibrissal control, although these have not yet been investigated.

If the precise positioning of vibrissae is important for efficient sensing, their flexible and long nature might, unfortunately, make them particularly prone to disruption, such as by strong winds, water movements or sound pressure waves. It is also worth bearing in mind that some species of cetaceans can use their vibrissal follicles (or crypts) to sense electromagnetic fields (Czech-Damal et al., 2012; Hüttner et al., 2022; Mynett et al., 2022). Indeed, findings from this Review suggest that vibrissae have the capacity to sense sounds, water movements, wind, electromagnetic fields, temperature as well as itch and pain (Czech-Damal et al., 2012; Dehnhardt et al., 1998; Efron et al., 2025; Hanke et al., 2010; Khasabov et al., 2020; Mugnaini et al., 2023). Furthermore, beyond its traditional role as a tactile sensory system, the vibrissal system of mammals engages tactile, olfactory, visual and auditory brain areas in a multimodal manner during active exploration (Allen et al., 2017; Efron et al., 2025; Parabucki and Lampl, 2017; Sieben et al., 2013). Being such a multimodal sense might make vibrissae particularly sensitive to environmental change, as they are likely to be affected by almost any perceptual alteration in the environment, including differences in sound, chemicals, wind, water movements, turbidity, temperature and electromagnetic fields, rather than just the vibrotactile pollution that we might expect to affect vibrissal sensing. Halfwerk and Slabbekoorn (2015) propose that one of the most important questions in the field of environmental change concerns the possible impacts of anthropogenic activities that emerge only when sensory pollution is multimodal. Most anthropogenic activity is multimodal; for example, traffic noise involves light, acoustic and chemical sensory pollutants. The vibrissal system, being inherently multimodal, could therefore provide a useful model system from which to better understand the impacts of multimodal pollution and environmental change.

Despite the important role that environment and ecology has on vibrissal form and behaviour, there is very little literature focusing on how environmental factors effect vibrissae. This is in contrast to other sensory systems that have been well studied; for examples, I refer the reader to recent reviews on the effects of acoustic pollution on hearing (Arcangeli et al., 2023; Engel et al., 2024), chemical pollution on olfaction (Wei et al., 2021) and light pollution on vision (Burt et al., 2023; Stöckl and Foster, 2022). Therefore, the aim of this Review is to identify environmental factors that are likely to affect vibrissal sensing. It will start by (i) defining relevant environmental change factors and (ii) establishing the sensory modalities involved in vibrissal sensing. It will go on to (iii) discuss how vibrissae may be affected by changing environmental stimuli; in particular, which sensory modalities. This is the first Review to place vibrissal sensing research within an environmental context, examining how environmental factors influence vibrissal form and function. It highlights a previously unexplored research area with potential implications for mammalian conservation, especially as the environmental change effects of vibrissal sensing may be impacting the foraging efficiency and health of keystone species, such as pinnipeds. The findings of this Review are reported narratively, and follow the Preferred Reporting Items for Systematic Reviews and Meta-Analyses (PRISMA) guidelines (sections Methods, Results and Discussion).

Glossary **Anticoagulant rodenticides**

Rodent pest control that prevents blood clotting, leading to internal bleeding.

**Barrel cortex**

Region of the primary somatosensory cortex (layer IV) that contains the barrel field, a topographic map of the facial vibrissae. Present in some species, including rats and mice.

**BOLD signal**

Blood oxygen level dependent signal, a measure of brain activity.

**Borna disease virus**

Severe neurological illness, mainly affecting horses and sheep. The disease is characterised by ataxia and abnormal depressive behaviour, frequently culminating in death.

**Brainstem**

Brain area situated at the base of the brain.

**Dermal papillae**

Projections of the dermis crucial for hair follicle function, particularly in controlling hair growth cycles and hair follicle development.

**Facial vibrissae**

Specialised stiff hairs, or whiskers, found on the faces of most mammals, including around the cheeks, mouth, eyes and chin.

**Far-field noise**

The sound produced by a source that is far enough away that it can be approximated as a point source.

**Herpes simplex virus**

A common viral infection that causes blisters or ulcers, often in the mouth.

**5-Hydroxytryptamine**

Serotonin, a neurotransmitter and hormone.

**5-Hydroxytryptophan**

Dietary supplement, converted to serotonin. Treatment for depression, migraines and weight loss.

**Intergovernmental Panel on Climate Change**

An intergovernmental body of the United Nations, which provides scientific information to develop climate policies.

**Intergovernmental Science-Policy Platform on Biodiversity and Ecosystem Services**

An intergovernmental organisation established to improve communication between science and policy on issues of biodiversity and ecosystem services. Similar to the IPCC.

**Methylphenidate**

A drug to treat attention deficit hyperactivity disorder and narcolepsy.

**Mystacial vibrissae**

Whiskers on the mystacial region of the face, where a moustache would be on a human (above the mouth and towards the cheeks).

**Neurotoxicity**

Harmful effects of toxic substances on the nervous system, including the brain and peripheral nerves.

**Organochlorine pollutants**

A group of persistent, synthetic chemicals containing chlorine, often used as pesticides or industrial products. Examples include DDT and PCBs.

**Oxidative stress**

An imbalance between production and accumulation of oxygen reactive species (ROS) in cells and tissues, and the ability of a biological system to detoxify them.

**Pure tone**

Sound with a sinusoidal waveform – a sine wave of constant frequency, phase shift and amplitude.

**Retinoic acid**

A naturally occurring metabolite of vitamin A.

**Somatosensory cortex**

Cortical region responsible for processing touch, vibration, pain, temperature and pressure.

**Thalamus**

Middle area of the brain that processes and distributes sensory and motor information to the cerebral cortex.

**Vibrissal crypts**

Remnants of vibrissal follicles on some species of dolphins, associated with electrosensing.

**Vibrissal placement response test**

Assesses an animal's ability to place a forelimb in response to whisker (vibrissal) stimulation.

**Vibrissal shafts**

The hair shafts of vibrissae or whiskers.

**Whisker specialists**

Certain mammal species that have developed and specialised whisker systems used for sensing their environment.

## Methods: environmental factors and article screening

### Environmental factor assessment

**Fig. 2.**
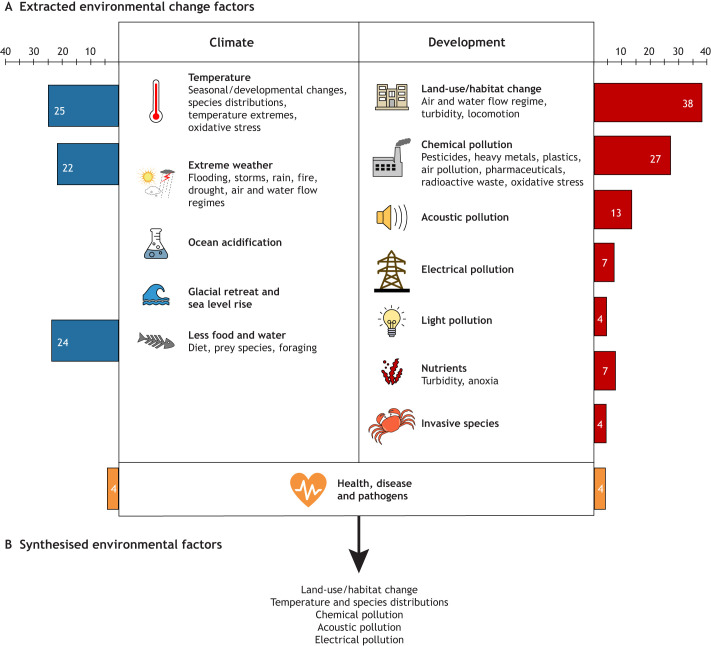
**Summary of environmental change factors.** (A) Extracted environmental change factors gathered from studying the latest reports by the Intergovernmental Panel on Climate Change (IPCC) and the Intergovernmental Science-Policy Platform on Biodiversity and Ecosytem Services (IPBES), alongside recent reviews. Aspects of environmental change can be thought of as being driven mainly by climate or human development processes, with health, disease and pathogens overlapping the two. The number of shortlisted vibrissal-sensing papers assigned to each category is shown in the bar charts, out of a total of 126. Approximately 40% of papers (49) were assigned to more than one category. (B) Following synthesis of the papers, this review will focus on the environmental factors: land use and habitat change, temperature and species distributions, chemical pollution, acoustic pollution and electrical pollution.

Environmental factors associated with global change were identified using the latest reports from the Intergovernmental Panel on Climate Change (IPCC; see Glossary) and the Intergovernmental Science-Policy Platform on Biodiversity and Ecosytem Services (IPBES; see Glossary) (Brondízio et al., 2019; Calvin et al., 2023), as well as recent, aligned reviews on other species, including birds (Richard et al., 2021), fish (van der Sluijs et al., 2011), insects (Yang et al., 2021) and humans (Sharma et al., 2023). Fig. 2 displays the environmental factors identified from these sources. Specifically, environmental change factors can be divided up into two main drivers: climate and anthropogenic development, with 13 environmental factors associated with them (Fig. 2). This includes temperature, which affects seasonal and development changes too (Brondízio et al., 2019). Temperatures are getting more extreme (Calvin et al., 2023), which may also cause oxidative stress (see Glossary) and neurotoxicity (see Glossary) (Sharma et al., 2023). Extreme weather events, such as flooding and storms, will affect air and flow regimes. Altered air and flow regimes will also be caused by land use and habitat change (van der Sluijs et al., 2011). Electrical and acoustic pollution (e.g. vibrations) may directly affect vibrissal sensations. Land-use change and increased nutrients can all increase water turbidity (van der Sluijs et al., 2011). Chemical pollution, disease and pathogens may all affect neurological processing and animal health. Food, drought, ocean acidification, glacial retreat and sea level rise are additional aspects of environmental change (Brondízio et al., 2019; Calvin et al., 2023) that will also be considered here. The shortlist of environmental factors (Fig. 2) was checked and confirmed by a specialist in environmental change, and all 13 factors were incorporated into subsequent literature searches.

### Literature searches and screening

Literature searches were conducted in the databases Scopus and Medline (PubMed) on 6 March 2025. ‘Whisker’ is a general term in engineering, chemistry and material science for a long, thin fibre, so searches were designed to exclude these topics and focus on sensory vibrissae. Specifically, the title and abstract were searched for three filters. The first filter consisted of vibrissa terms: (vibriss* OR ‘tactile hair’) OR [whisker AND (sens* OR feeling OR perception)]. The second filter included the environmental factors identified from Fig. 2 and the third filter included NOT terms to remove some of the engineering and material science literature. The full search criteria can be found in the Supplementary Materials and Methods. This led to 2008 articles in Scopus and 995 articles in Medline, which were uploaded for screening into Rayyan (Ouzzani et al., 2016); 456 duplicate articles were removed, and 2537 articles were screened, firstly using just the abstracts (*n*=2537) and then full text (*n*=170). Screening involved excluding articles based on the criteria in Table 1; namely, that it was not a suitable article type, did not contain animal vibrissae, did not report relevant data or did not use a relevant environmental stimulus. A full PRISMA diagram is included in Fig. S1, to summarise this process. This led to 126 articles being included for extraction.

**Table 1.** Summary of the article screening and extraction process: exclusion criteria

Total no. excluded	Label	Description
44	Article type	Not original data article, i.e. review, perspective, editorial.
559	Not animal facial vibrissa	Excluded examples include artificial whiskers (although some closely bio-inspired examples with biological implications were included), engineering material fibres, animal studies without vibrissae, not facial vibrissae (i.e. body or carpal vibrissae), an extinct species.
285	Not relevant data	Vibrissal responses not measured, focused on other aspects instead, i.e. anatomy description only, modelling only, methods-based, validation paper.
1522	Not relevant environmental stimulus	Not a natural environment cue, study focused on optogenetics, genetically engineered mice, mouse models, social cues, stem cells, general stress, general pain, injury, stroke, brain chips, ageing, development, hair loss treatments, learning, memory, alcohol, chemicals not found in the environment, whisker trimming, anaesthesia, no environmental cue.

In the extraction process, the full text of each article was reviewed to identify the species involved and the main findings, and also to allocate the paper to a relevant environmental factor (Fig. 2, with details in Table 2). The full coding and summary of all papers can be found in Table S1. For the structuring and synthesis of the narrative of this Review, some of the environmental factors were combined for reporting. Land use and habitat change, weather, light and nutrients were all grouped under the heading ‘land-use and habitat change’. Temperature, invasive species, health, disease and pathogens and diet were all grouped under the heading ‘temperature and species distributions’, and chemical pollution and dietary chemicals from isotope analysis were grouped under ‘chemical pollution’. Acoustic and electrical pollution were reported individually.

**Table 2.** Summary of the article screening and extraction process: environmental factor scoring criteria

Grouping for narrative	Label	Description
T	Temperature	Including: temperature, daily and seasonal effects, growth and moult, changing species distributions, itch and allergies
T	Invasive species	Invasive plants
T	Health, disease and pathogens	Tumours, viruses
LH	Weather	Air and water flow regimes, hydrodynamics
T, C	Food and diet	Foraging behaviours and sensing techniques (T), specific prey species (T), isotope analysis (C)
LH	Land-use and habitat change	Enriched environments, movement around habitat, air and water flow regimes, hydrodynamics, turbidity, visual loss, vision
LH	Light pollution	Light
LH	Nutrients	Turbidity, visual loss, vision, anoxia
C	Chemical pollution	Chemicals that can be found in the environment, drugs, pharmaceuticals, vitamin A, metals, carbon dioxide, odours
A	Acoustic pollution	Audition, sound, including wind noise
E	Electrical pollution	Electromagnetic fields, radiofrequency, static electricity
–	Not observed	Ocean acidification (OA) and glacial retreat (G), less water

Narrative groupings are indicated as: T, temperature and species distributions; LH, land-use and habitat change; C, chemical pollution; A, acoustic pollution; E, electrical pollution.

## Results: species and sensory modalities

**Fig. 3.**
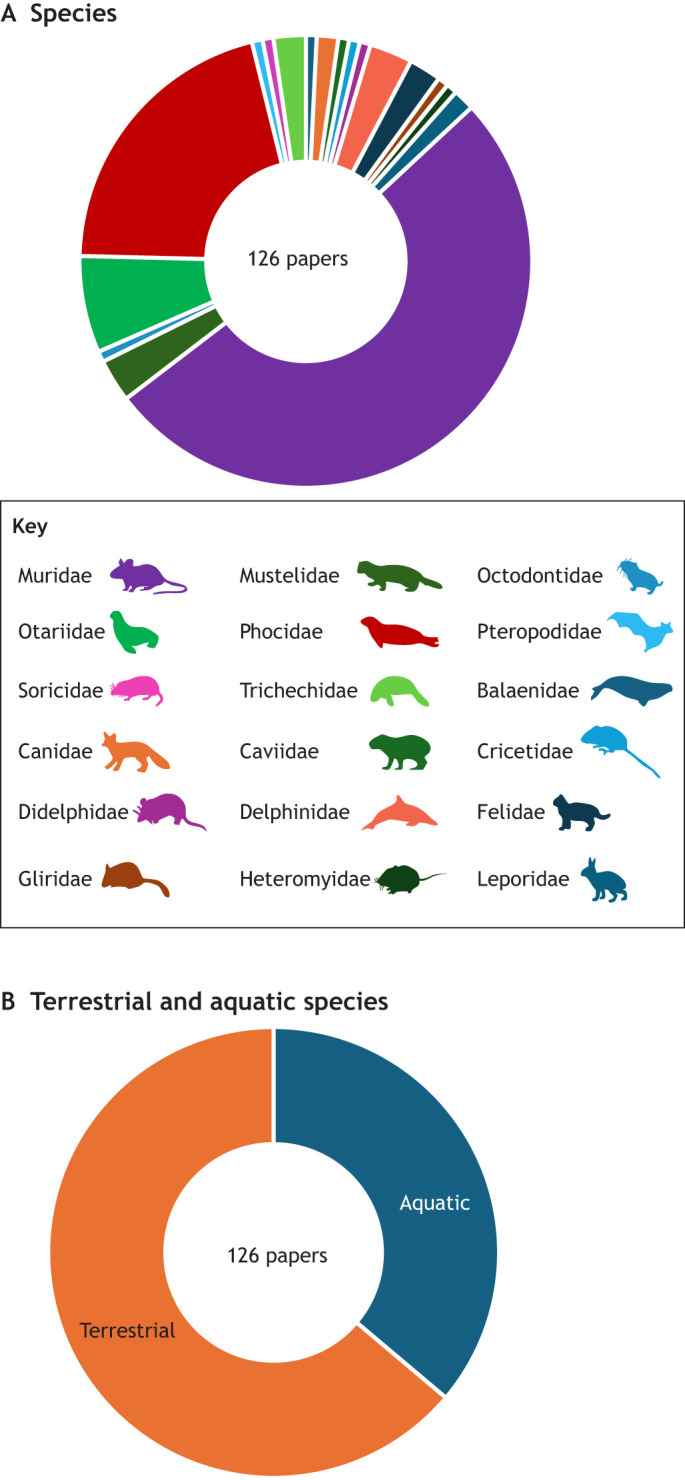
**Summary of species studied in the shortlisted papers.** The majority of studies were conducted on species of Muridae (52%), which mainly included laboratory mice (18%) and rats (32%), and Phocidae (21%), which mainly included harbour seals (16%). Therefore, the majority of the studies were on terrestrial species (64%), rather than aquatic (36%).

In the 126 shortlisted papers for extraction, most of the studies used laboratory rats (32%) and mice (18%); in fact, 52% of all studies involved murids (Fig. 3A), and 64% of the studies were based in the terrestrial environment, rather than in the aquatic environment (36%) (Fig. 3B). Of the aquatic species, the phocidae (21%) and especially harbour seals (16%), were the most well represented (Fig. 3A). There was a total of 36 species studied overall, including whales, dolphins, sea lions, cats, foxes, dormice, manatees and shrews, to name but some examples (Fig. 3A).

Of the environmental factors, land use and habitat change included the most papers (21%), followed by chemical pollution (15%), and then temperature (14%), weather (14%) and food and water (14%) (Fig. 2). No papers were aligned to the factors of ocean acidification, glacial retreat, sea level rise and shortages of drinking water (Fig. 2, Table 2).

It is most common to study vibrissae from the perspective of touch sensing (see recent reviews in rodent neuroscience for more information on this topic: Campagner et al., 2018; Diamond et al., 2008; Evans et al., 2019; Prescott et al., 2011). However, touch sensing studies were relatively underrepresented by papers in this literature review this literature review (19 papers, 15%), as the majority of touch sensing papers used irrelevant environmental stimuli, such as artificial poles, shapes and textures, rather than realistic environmental stimuli or more realistic environments. However, the literature search here did identify studies that considered vibrissal sensing across an array of multimodal applications, including sensing wind and water movements, sound, electromagnetic fields, temperature and itch and pain, which will be considered in turn below.

### Sensing wind and water movements (24 papers, 19%)

In the terrestrial environment, rats use their vibrissae to locate air movements, and they tend to turn towards the direction of the flow (Yu et al., 2016a,b). The supraorbital (above the eye) and the more caudal and dorsal mystacial vibrissae (see Glossary) are all likely to sense wind (Mugnaini et al., 2023). They bend in the direction of airflow and how much they bend increases with flow speed (Yu et al., 2016a,b). When rats move their vibrissae during a task, as they would naturally do, they become worse at locating air movements (Ollerenshaw et al., 2012), which means that self-movement introduces noise to the system.

In the aquatic environment, water rats, phocid seals, otariids and manatees can all sense hydrodynamic signals from water movements, probably to help guide their foraging (Adachi et al., 2022; Gläser et al., 2011; Hanke et al., 2010; Reep et al., 2011). These signals can be small and specific, even below the usual hearing ranges of these animals (Gaspard et al., 2013; Murphy et al., 2017). Harbour seals can detect stimuli that are representative of flatfish breathing, as well as the glide phases from fish swimming (Niesterok et al., 2017; Wieskotten et al., 2010). The vibrissae of North Atlantic whales are a similar size to plankton and are thought to be able to detect the tiny water movements from their plankton prey (Murphy et al., 2022). Both harbour seals and California sea lions can also follow flow trails (Gläser et al., 2011; Schulte-Pelkum et al., 2007). Harbour seals, and many other phocid seals, have undulating vibrissae, which reduce vortex-induced vibrations, almost 10 times more than non-undulating vibrissae (such as those of otariids, cetaceans and manatees) (Hanke et al., 2010). Like rats, harbour seals tend to orient towards the flow direction, which reduces vibrations of their vibrissae; however, flows that hit the vibrissae at 60–90 deg cause instability and oscillations of the vibrissae (Wang and Liu, 2017).

### Sensing sound (13 papers, 10%)

Acoustic and somatosensory signals are relatively well coupled in rodents. Stimulating the vibrissae causes activity in their auditory cortex (Lohse et al., 2021). There is also a polysensory area between the primary somatosensory cortex (see Glossary) and the primary auditory cortex in rodents, which responds to both sound and touch (Di et al., 1994). A small population of neurons in the secondary somatosensory cortex in mice respond only to sound (Zhang et al., 2020). When mice move their vibrissae against surfaces, it produces audible sounds that fall within their hearing range and are detected by the auditory cortex (Efron et al., 2025). Furthermore, when sensations from the vibrissae are abolished, mice can still identify objects, relying only on the auditory stimulus (Efron et al., 2025). Therefore, it is likely that the vibrissal system engages both auditory and tactile modalities, which may suggest, in rats and mice at least, that sound will impact vibrissal sensing. The soundwaves themselves can also move vibrissae in rodents. In rats, audible sound from a speaker (at 60–180 Hz) moved the vibrissae a similar amount to cricket cercel hairs or inner ear hair bundles (Shatz and Christensen, 2008). In mice, facial motion next to the vibrissae was 30 dB more sensitive to sound than using an acoustic startle response (Clayton et al., 2024), suggesting that sound may be detected by both the vibrissae and the surrounding skin.

In grey seals, tactile signals have been found to only be detected by tactile regions of the brain, and auditory signals by the auditory part of the brain, suggesting local responses to each sense in the phocid brain (McKnight et al., 2021). However, phocid vibrissae do move in response to sounds, although their undulations supress far-field noise (see Glossary) and wind sounds by 10–13 dB in harbour seals (Chen et al., 2025; Liu et al., 2022). Harbour seal vibrissae vibrate over a broad range of frequencies (20–250 Hz), which is larger than the usual range of water movements (Murphy et al., 2017). Their ability to localise sounds is also very good, despite their small pinnae (Byl et al., 2016). This suggests that, despite dampening acoustic signals, pinnipeds might well be able to use their vibrissae for sensing acoustic cues. Saimaa ringed seals have a distinct fatty acid composition around their vibrissal follicles, which is different to that of blubber (Käkelä and Hyvärinen, 1993). It has been suggested that this is not a function of diet, but may serve to translate sound signals and improve acoustic sensing using the vibrissae (Käkelä and Hyvärinen, 1993).

### Electric sensing (7 papers, 6%)

Rats, bottlenose dolphins and Guiana dolphins have all been found to respond to electric fields using their vibrissae, or vibrissal crypts (see Glossary) (Czech-Damal et al., 2012; Hüttner et al., 2022, 2023; Weigel and Lundstrom, 1987). In terrestrial mammals, electric fields, such as static electricity, can cause movement of the vibrissae in rats, which disappears at high levels of humidity (>40%) (Weigel and Lundstrom, 1987). However, introducing electromagnetic field and radiofrequency disturbance (such as that emitted from conventional wifi devices) does not affect vibrissal behaviours in rats (Anselmo et al., 2006; Othman et al., 2017) or neural responses during vibrissa stimulation in mice (Orlacchio et al., 2022). Therefore, it is unlikely that current levels of electrical pollution are affecting small terrestrial mammals. In the aquatic environment, both bottlenose dolphins and Guiana dolphins respond behaviourally to electric fields, using their vibrissal follicles, to a similar detection threshold to that of platypus (0.5–5.5 µV cm^−1^) (Hüttner et al., 2022). They probably use this sense for finding prey in sediment, such as during crater feeding (Czech-Damal et al., 2012). As electric sensing has been found in the two delphinid species that have been tested so far, it might also be present in other aquatic species (Mynett et al., 2022).

### Temperature sensing (8 papers, 6%)

Both rats and rabbits have temperature sensors around their nose, whisker pad and mouth (Dickenson et al., 1979). Spix cavy, harbour seals and Guiana dolphins all have high surface temperatures at the opening of the vibrissal follicle, compared with that of the surrounding skin (Mauck et al., 2000; de Queiroz et al., 2020). This suggests that the vibrissae have an independent blood supply that may well be thermoregulatory (Mauck et al., 2000). The majority of previous research has focused on cold temperatures, with vibrissae seemingly well adapted to cold conditions. Both rats and rabbits have more cold sensors around the vibrissae than warm ones, which are also mapped topographically in brainstem areas (Dickenson et al., 1979). Cats have been noted to have vibrissal reflexes at cool temperatures (Britton, 1922). Harbour seals and Guiana dolphins probably heat their vibrissal follicles at cold temperatures (Erdsack et al., 2014; Mauck et al., 2000). Indeed, harbour seals have a three-part follicle and high hair densities around the follicles to help keep them warm (Erdsack et al., 2014). Their vibrissae are also as sensitive in cold water as in warm water as a result of the follicle maintaining the warmth and sensitivity of the mechanoreceptors at cold temperatures (Dehnhardt et al., 1998).

The warmer temperature of the follicles compared with the rest of the skin, which has been observed in many species, may suggest that the vibrissal region acts as a thermal window for radiative heat reduction (de Queiroz et al., 2020). Undulating phocid vibrissae also help to transfer heat from the surface of the vibrissa into the water (Yoon et al., 2020). This suggests that vibrissae may have some useful adaptations for reducing internal heat in warming environments.

### Sensing itch and pain (1 paper, 1%)

Itch and pain are likely to be coded for by vibrissal areas in the brain, as histamine activates lots of neurons in the vibrissal barrel fields (Khasabov et al., 2020), although how this impacts vibrissal sensing is unknown.

### Dietary specialisations of the vibrissal system (25 papers, 20%)

**Fig. 4.**
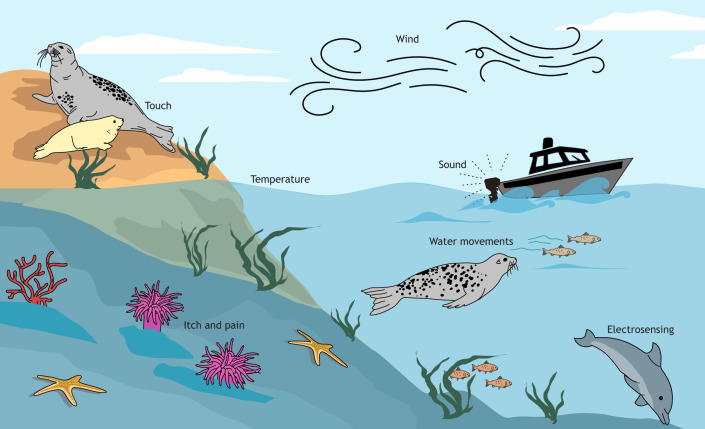
Summary of literature review findings illustrating the multimodal nature of vibrissal sensing in the environment.

Overall, the studies from this literature review emphasize the multimodal nature of vibrissal sensing (Fig. 4). The vibrissal system has been identified here as being involved in sensing touch, sound, wind, water movements, electrical fields, itch, pain and temperature (Fig. 4). Functionally, vibrissae have been identified as being used for social interactions (Blanchard et al., 2001; Wolfe et al., 2011) and to guide orientation and locomotion within the habitat, including identifying stimuli within the surrounding space (Arkley et al., 2014, 2017; Efron et al., 2025). However, perhaps the greatest focus of studies has been on how vibrissae guide foraging in dark habitats, such as at night or underwater.

Harbour seal vibrissae can detect the exact hydrodynamic signatures of their prey, including breathing and swimming styles (Niesterok et al., 2017; Wieskotten et al., 2010). Guiana and bottlenose dolphins can detect the electrical fields of their prey while foraging in sediment-rich environments (Czech-Damal et al., 2012; Hüttner et al., 2022), and North Atlantic right whales have vibrissae positioned frontwards and sized at the same scale as their plankton prey (Murphy et al., 2022). Other specialised examples also exist. For example, Etruscan shrews use their vibrissae to direct precise attacks on cricket prey items by localising the spines on their back legs (Anjum et al., 2006). The plains viscacha rat has in-growing vibrissal hairs in its mouth, behind its incisors, that vibrate using their extrinsic vibrissal musculature to strip leaves (Berman, 2003). Nectar feeding bats have longer vibrissae than non-nectivarious bats, which help to guide hovering positions during feeding (Amichai et al., 2023). Galapagos sea lions that forage benthically on cusk eels use their vibrissae in the sediment, which wears the vibrissae and makes them shorter than those of pelagic foragers, which have much longer vibrissae (Schwarz et al., 2022). The length of the vibrissae can even be used to group individuals by their primary foraging method (Schwarz et al., 2022). This foraging polymorphism reveals a flexibility in foraging behaviours that could help to protect the sea lions against the negative effects of climate change; however, it is unlikely to be enough to reverse their ongoing population declines caused by anthropogenic changes in the environment (Schwarz et al., 2022).

## Discussion: effects of environmental change

**Fig. 5.**
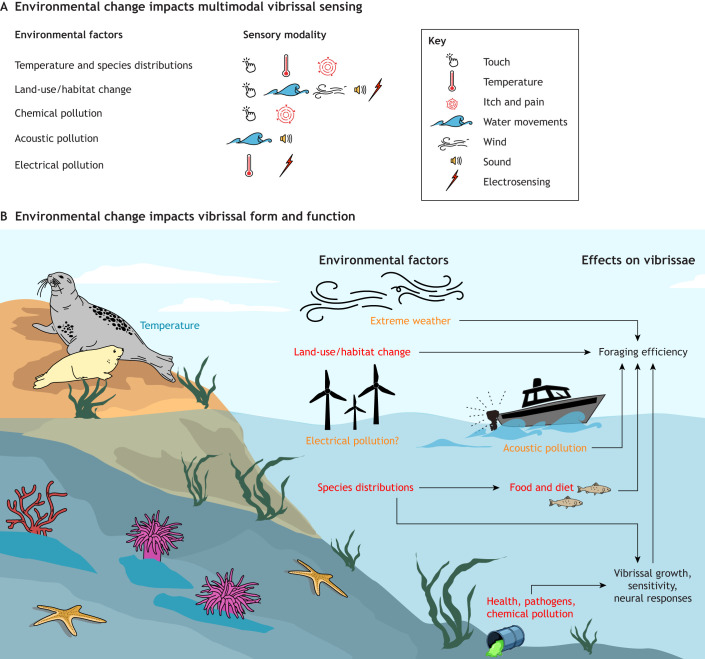
**Summary of literature review findings illustrating how environmental factors might be affecting vibrissae.** (A) The multimodal effects of the environmental change factors, and (B) how the factors affect vibrissal growth, sensitivity, neural responses and foraging efficiency. Vibrissae appear to be relatively robust to changes in temperature, and the effect of electrical pollution remains relative unknown. However, additional water and air movements, and vibrations that arise from acoustic pollution, land-use change and extreme weather may affect foraging success. In addition, species distributions, pathogens and chemical pollution may disrupt vibrissal growth, sensitivity and neural responses, which would affect all elements of vibrissal use. Factors are colour coded using a combination of the number of reported studies and the risk to vibrissal sensing, with blue being relatively robust to change to that factor, red being a cause for concern and orange being intermediary.

Armed with a better understanding of the vibrissal system, and its multimodal sensory abilities, the impact of environmental change on vibrissal sensing is now considered. Because of the broad perceptual abilities of vibrissae, this may leave them open to many factors of environmental change, across a broad range of modalities (Fig. 5A).

### Land use and habitat change

Land-use changes include an increase in crops and pastures, as well as urbanisation and infrastructure expansion (Brondízio et al., 2019), perhaps suggesting that environments are becoming more uniform, with less complete and structurally complex vegetative cover. Overall, rodents exposed to enriched, more complex environments have higher, quicker neural responses to vibrissal touches than those from normal or impoverished environments (Devonshire et al., 2010; Seo, 1992), although much of this work has only been undertaken in laboratory environments. Rodents also make larger vibrissal movements in response to changing and more complex environments (Arkley et al., 2014). Therefore, more uniform environments may impact both vibrissal movements and neural responses. Because of fragmentation of the landscape, animals may also have limited capacity to move around between suitable habitats. Laboratory studies have found that natural locomotion during vibrissal sensing helps to increase neural activity, including the barrel cortex (see Glossary) activity and the BOLD (blood oxygen level-dependent) signal (see Glossary), in response to vibrissal touch (Ayaz et al., 2019; Eyre et al., 2022). Indeed, naturally, small quadrupedal mammals use their vibrissae to guide safe forepaw placement during locomotion (Grant et al., 2018), especially during climbing. Gaps in their habitat, beyond a vibrissa reach, can affect small mammals; for instance, European dormice will travel more on the floor and eat less when there are gaps in their canopy beyond what they can reach with their vibrissae (Arkley et al., 2017). Observations of vibrissal use in rodents reveals specific adaptations to their natural habitats. For instance, rats foraging in tunnels will meet head-on with whisker–whisker touches; following this, submissive individuals will be able to turn away dominant individuals from their tunnel (Blanchard et al., 2001). Dominance hierarchies and social interactions could well be altered in more open environments.

Changes in land use and habitat can affect how air moves around the terrestrial environment, and how water moves around the aquatic environment (van der Sluijs et al., 2011). Extreme weather events, such as storms, will also increase wind and water movements. Changing these flow regimes around the environment will introduce noise to vibrissal sensing, including vibrotactile and acoustic noise, as well as increased visual motion (Halfwerk and Slabbekoorn, 2015), although this has never been studied before. Changing land use and habitats can also impact sediment distribution and nutrient cycling in the aquatic environment, increasing the turbidity of the water and reducing an animal's reliance on vision. For example, deforestation, mining and other anthropogenic development activities upstream can result in increased sediment loading, which makes water cloudy. Whisker sensing could easily help compensate somewhat for vision. In animals (such as opossums or rats) that have been blind from a young age, some compensatory mechanisms can be seen in the neural processing of vibrissal touch signals and their vibrissae tend to be more sensitive (Abe and Yawo, 2018; Ramamurthy and Krubitzer, 2018). However, these mechanisms are not present if blindness occurs at later ages, suggesting that extreme turbidity that occurs for short periods, such as from extreme weather events, will not initiate compensations, especially in adult animals. Visual and touch signals occurring together, such as those that arise during locomotion and head rotations, causes increased signals in the somatosensory cortex and thalamus (see Glossary) (Allen et al., 2017; Sieben et al., 2013), suggesting that vision and touch usually work best together. Indeed, while vibrissae perform well in dark environments, integrating with vision is likely to improve their performance.

### Temperature and species distributions

Increasing temperatures are affecting seasons, species distributions and ultimately circadian day–night cycles too. The skin around the vibrissae in mice is sensitive to light and entrains to light–dark cycles (Buhr et al., 2019). Responses in the mouse barrel cortex also change daily, where in the dark (active) part of the night, there are more inhibitory synapses on the dendritic spines of neurons, and in the light (rest) phase of the day, there are more excitatory synapses. These patterns are likely to shift, if light and night–day timings also change, as a result of both changing seasons and light pollution. Similarly, seasonality may affect vibrissal moults. Warm winters and springs are affecting moulting in Baikal seals (Petrov et al., 2021) and it is likely that whisker renewal, growth and replacement will be affected by changing seasons.

Increasing temperature affects both seasonality and species distributions. This means that whiskered animals may find themselves in different environments, and exposed to different plant and animal species, including new prey items. Laboratory studies have found that some extracts from plants, algae and fungus increase vibrissal shaft growth and dermal papillae (see Glossary) cell growth within the follicle (Kang et al., 2013b; Matsuda et al., 2003; Rho et al., 2005; Sakaguchi et al., 2004; Sun et al., 2013; Wang et al., 2023), including extracts from invasive species commonly found in the environment, such as *Polygonum multiflorum* (a plant of the buckwheat family) and wheat bran (Kang et al., 2013a; Sun et al., 2013). Increasing the length of the vibrissal shaft increases the bending moment at the base (Dougill et al., 2020), which will impact the forces transduced into the follicle, and ultimately the sensation of touches.

Seasonal changes are altering diets and food availability. The offspring of rats fed on a high fat diet had their vibrissal placement responses delayed by over a week in development (Giriko et al., 2013). A diet high in magnesium causes mineralisation of the vibrissal capsule in mice (Kupetsky-Rincon et al., 2012) and a diet low in copper can cause impaired vibrissal guided foot placement in rats (Pyatskowit and Prohaska, 2008). We have also already heard about how specialised vibrissae are for foraging. With prey and plant distributions varying, preferred food types might not be available and specialised vibrissal adaptations will be less advantageous. Foraging success may even be affected in some of these species with specialised vibrissae.

Species distribution changes and increasing temperatures can also affect disease and pathogen exposure. This has rarely been studied with respect to the effect of whisker sensing, although different viruses are likely to impact the vibrissae in specific ways. For example, Herpes simplex virus (see Glossary) delivered via a lip scratch caused vibrissae to become less responsive to touch in the cotton rat (Boukhvalova et al., 2020), whereas neonatal Borna disease virus (see Glossary) did not affect vibrissal sensing in lab rats (Pletnikov et al., 2001). Because of the specialised anatomy of the vibrissae follicles, tumours and viruses might affect these areas differently, compared with other tissues, such as the skin. For example, hair follicle tumours have different histological structures when they are around tactile hairs of house musk shrews, compared with the skin of the head, face and neck (Kimura, 2022). Furthermore, the rabies virus antigen can be found in the outer root sheath of the vibrissal follicle in dogs, suggesting that vibrissae might be a useful tissue to use for diagnosing disease (Shimatsu et al., 2016).

### Chemical pollution

The vibrissal shaft stores chemicals, as well as disease markers. Therefore, vibrissae are often plucked and analysed for chemical analysis to study pollutants and species diets. What this means for the functioning of vibrissae or the health of the individual is unknown. It may indicate that contaminants can collect in the inert whisker tissues, where they cannot cause toxic effects (White et al., 2025). Anticoagulant rodenticides (see Glossary) have been found in the vibrissae of polecats (Sainsbury et al., 2018), organochlorine pollutants (see Glossary; persistent organic pollutants, POP) have been found in the vibrissae of fur seals (White et al., 2025), vitamin A in the vibrissae of mice (Vanexan and Hardy, 1979), and trace elements in the vibrissae of fur seals and sea lions (Kooyomjian et al., 2022). Because vibrissae accrue chemicals, signatures of carbon and nitrogen from them can be used to identify diet signatures that can be tracked over time, as well the temperatures and types of environments that the species have been foraging in (Elliott Smith et al., 2025; Tucker et al., 2024; Walters et al., 2020). This has been successfully trialled in Peruvian fur seals, Antarctic fur seals, California sea lions, grey seals, bearded seals, Weddell seals, sea otters, sea lions, American mink, cat and island fox (Calvo-Mac et al., 2024; Edwards et al., 2021; Goetz et al., 2017; Lerner et al., 2018; Newsome et al., 2009; Page et al., 2021; Rosas-Hernández et al., 2018; Tyrrell et al., 2013; Walters et al., 2020). Tracking chemical accumulation over time can only work if the growth of the vibrissae can be predicted, which might be altered by seasonal changes in the environment.

Laboratory studies have found that some chemicals that occur in the environment can also cause heightened activity in areas of the brain that process vibrissal signals, including vitamin B12, 3,4-methylenedioxymethamphetamine (MDMA), heavy metals and cocaine (Kang et al., 2019; Papp et al., 2006; Pecze et al., 2005; Rutter et al., 2005). Conversely, some chemicals, including retinoic acid (see Glossary) and methylphenidate (see Glossary), reduce neural responses (García-Fernández et al., 2000; Starr et al., 2008), and carbon dioxide has no effect (Tournissac et al., 2024). Increased responses may not necessarily be good. Increasing neural responses to small magnitude deflections of the vibrissae may enhance detection of a movement, but impede discrimination of the event (Rutter et al., 2005). Indeed, any change in usual responses to whisker sensing may indicate a distortion of the signal (Starr et al., 2008).

Chemicals can also impact the vibrissae more directly. Molnupiravir (an antiviral medication) can cause broken vibrissae in cats (Roy et al., 2022), and tyrosine kinase inhibitors (often used to treat cancer) causes irregular growth of the vibrissae, with the thickness and curvature of the vibrissae also looking altered (Zhang et al., 2021). 5-Hydroxytryptophan (5-HTP; see Glossary) and 5-hydroxytryptamine (5-HT, serotonin; see Glossary), produced for dietary supplements, cause rapid whisker movements in rabbits (Banerjee et al., 1970). Behavioural responses may also occur in response to the smell, or odour, of certain chemicals. For instance, ammonia causes vibrissal protraction in rats (Callado Pérez et al., 2023), which is indicative of a grimace. Banana and pineapple smells both cause changes in vibrissal positions and movements (Renard et al., 2022). Indeed, breathing and whisker movements are coupled in rodents and controlled by the same pattern generator in the brainstem (Kleinfeld et al., 2015). Vibrissal stimuli are also detected in the olfactory bulb, which is correlated with barrel cortex activation in rats (Parabucki and Lampl, 2017), suggesting that chemosensing and vibrissal sensing are well coupled.

### Acoustic pollution

The rise in noise pollution is mainly driven by human population growth and development, and anthropogenic noise is a major pollutant globally (Kok et al., 2023). The effect of acoustic noise on vibrissal sensing is rarely studied, making up only a tenth of the included studies in this literature review. However, acoustic pollution is likely to be felt by the vibrissae of both terrestrial and aquatic mammals. Indeed, mice receiving an audible auditory stress (3000 Hz noise of 90 dB for 24 h) reduced responses in the somatosensory cortex to vibrissal stimulation (Rezaei et al., 2021). Rat pups exposed to an audible pure tone (see Glossary; 4000 Hz, 64 dB for 8 h a day) had accelerated development of reflexes, including vibrissal placement responses (So et al., 2010).

### Electrical pollution

Electropollution, such as the electrical noise from power generators, is an environmental factor that can directly influence the transmission of electrical fields (van der Sluijs et al., 2011). We do not have a good understanding of the strength of electrical pollution in the aquatic environment, such as from undersea power cables from offshore wind farms, although researchers currently do not think that dolphins will be able to detect the reported ∼0.005 µV cm^−1^ amounts of electric field pollution in the environment (Taormina et al., 2018). However, this might change if electrical pollution continues to increase in the environment.

### Impact of changing environments on vibrissal sensing

Throughout this Review, examples have shown that vibrissal sensing is surprisingly robust to certain aspects of environmental change. Although it is not really possible to compare between different sensory modalities, it would be interesting to consider whether vibrissal sensing is more robust to environmental change than auditory, visual or chemosensory systems. Examples include vibrissal follicles aiding in thermoregulation and supporting cooling by providing thermal windows (Mauck et al., 2000; de Queiroz et al., 2020). The undulating vibrissae of phocid seals are especially resilient to change, conducting heat away from the skin, and also reducing noise for hydrodynamic sensing (Hanke et al., 2010; Wang and Liu, 2017; Yoon et al., 2020). Vibrissal sensing may also provide a compensatory sense when vision cannot be relied upon (Ramamurthy and Krubitzer, 2018), such as in turbid environments, caused by sediment and nutrient build-ups. Vibrissal tissues may even provide an inert storage area for chemicals (White et al., 2025). However, with climate and land-use changes occurring on an unprecedented scale, the extremes of temperature and effects of land-use change and pollution might yet have significant impacts on vibrissal sensing. Indeed, while vibrissal sensing may be relatively robust to changes in temperature, it is probably becoming increasingly vulnerable to a range of environmental changes driven by climate shifts, pollution and habitat alteration (Fig. 5). These environmental change factors cause multimodal alterations in the environment (Halfwerk and Slabbekoorn, 2015), which affects all the identified modalities of vibrissal sensing (Fig. 5A), as well as other important aspects of vibrissal from and function (Fig. 5B).

The movement of the vibrissae can be affected by changes in air and water flow regimes and acoustic noise (Chen et al., 2025; Liu et al., 2022; Shatz and Christensen, 2008), which may have the capacity to mask important stimuli, such as prey signals, although this has not yet been studied. This may be especially impactful as many natural hydrodynamic signals are minute, such as the hydrodynamic signals of plankton or the breathing of flat fish (Murphy et al., 2022; Niesterok et al., 2017). Indeed, there are some interesting specialisations of vibrissal form with diet and foraging techniques documented in this Review. These range from electrosensing the heartbeat of prey in sediment to having vibrating inner mouth bristles to strip desert leaves (Berman, 2003; Czech-Damal et al., 2012). Changes in the distribution of prey and forage species due to climate change are likely to affect foraging success in some of these species (Fig. 5B) but may also give rise to new adaptations in the future.

Coming into contact with new plant species as distributions change worldwide may cause allergic reactions, and extracts from these plants may alter the growth and shape of vibrissae (Kang et al., 2013b; Khasabov et al., 2020; Sun et al., 2013). Increases in diseases and pathogens have also occurred as a result of climate change and increased transport (Calvin et al., 2023), and many might affect vibrissal sensing, such as herpes virus, which alters the sensitivity of the vibrissae (Boukhvalova et al., 2020). Different chemicals have also been shown to broadly affect both vibrissal growth and neural responses following vibrissa stimulation (Papp et al., 2006; Pecze et al., 2005; Roy et al., 2022; Zhang et al., 2021). Because vibrissal shafts pass along vibrotactile information, their growth and length will affect how they bend and vibrate. Altering neural responses to vibrissal stimuli is also likely to distort vibrissal signals (Starr et al., 2008). Therefore, the effects of chemical pollution and changing species distributions, especially new plant species and pathogens, could be far reaching. They are likely to affect vibrissal sensitivity and signalling and have important implications for animal health (Fig. 5B). Conversely, rather than just considering how environmental factors affect vibrissae, the vibrissae themselves can be used to monitor the environment directly with isotope analysis, which has the capacity to identify disease, chemicals in the environment and diet shifts with food availability (Bodey et al., 2010; Kernaléguen et al., 2016; Newsome et al., 2009; Tucker et al., 2024). This is an emerging field in mammalian biology. If we can identify the relationship of vibrissal growth over time for each considered species (Greaves et al., 2004; Kernaléguen et al., 2016; McHuron et al., 2016; Robertson et al., 2013), vibrissae could act as exceedingly useful indicators of environmental chemical pollution.

### Future research recommendations

Many aspects of environmental change cannot be assessed just yet, as we do not know the full extent of the problem. Indeed, in this literature review, 64% of studies were from the lab, 14% from other captive collections and only 21% from the field in a true natural environment. Certainly, there are challenges in inferring field implications from laboratory studies. However, in order to truly examine how chemical pollution, flow changes and acoustic and electrical noise may impact vibrissal sensing, we need to better understand both the levels in the environment and the levels that will impact vibrissal responses and behaviour, which probably requires both field and controlled laboratory investigations. We have a limited understanding of the natural stimuli that the animals usually sense, such as the water signals from fish that can be used for hunting. Indeed, the majority of articles were rejected from this literature review because they did not have realistic and relevant environmental stimuli (60%, Supplementary Materials and Methods). Therefore, as well as understanding better the levels of pollution and noise in the environment, it is also imperative to design studies that incorporate relevant environmental stimuli to investigate their impact on vibrissal sensing.

The majority of species in the studies were laboratory rats and mice, featured in 50% of papers. Many previous studies on wild rats and mice, although beyond the remit of this Review, are focused on urban environments, which are expanding across the globe as a result of development and habitat change (Collins et al., 2021). Because many rodents use their whiskers as their primary sense (Grant and Arkley, 2016) and whiskers can be used as indicators of environmental stress (Fardell et al., 2021), perhaps studying the effect of urbanisation on rodent vibrissal sensing might be a useful first case study to bridge the gap between laboratory and field-based research.

Interesting species-specific adaptations in whisker use and sensing were also observed, especially in terms of foraging and feeding. Therefore, it is vital to open up vibrissal research beyond model animal species, such as rodents, to better understand these important species-specific associations. It is only with this in mind that we can better grasp how distributions of prey species and forage may affect feeding and survival in whiskered animals during periods of environmental change. While there could be many recommendations that arise from this Review, these two observations have both been clear from the start of this process, as soon as the literature was surveyed (Table 1, Fig. 2): (i) we need to develop studies with relevant environmental stimuli; and (ii) we need to study more diverse species.

To move the field forward, we must, therefore, bridge the gap between laboratory conditions and the complex realities of the natural world. Without realistic environmental stimuli and broader species representation, we risk missing the full picture of how environmental change truly impacts vibrissal sensing and, ultimately, mammalian survival.

## Conclusions

While vibrissae and vibrissal sensing demonstrate some resilience to environmental changes, increasing threats such as pollution (chemical and acoustic), altered species and pathogen distributions, and flow regime changes may significantly impair vibrissal function. These impacts could disrupt critical behaviours such as foraging and orientation, with broader implications for survival. Addressing these challenges will require researchers to understand and develop more ecologically realistic sensory stimuli and adopt a broader taxonomic focus in their work. With these two goals, we can start to understand the vulnerability and adaptability of vibrissal systems in a changing world. With environmental change factors being inherently multimodal, mammalian vibrissae provide a specialised sensory system from which to study the multimodal effects of environmental change, providing an integrated approach from which to explore the full ecological impact of human activities on mammalian sensory performance and perception. This has especially important implications for the conservation of charismatic, keystone mammalian whisker specialists, such as pinnipeds and sea otters, to name but a few.

## Supplementary Material

10.1242/jexbio.250776_sup1 Supplementary information
