# Supplementary material for: Vibrissal sensing in mammals in a changing world
Source: J Exp Biol. 2026 Feb 11;229(Suppl 1):jeb250776. doi: 10.1242/jeb.250776 (PMC12952711; doi:10.1242/jeb.250776)
Supplement: Supplementary information [file jexbio-229-250776-s1.pdf]

Supplementary Materials and Methods

Literature searches and screening records

- Open science framework registered on 6th March 2024
- 6th March 2025 all searches conducted

Overall title, abstract and keywords were searched for:

vibriss* OR “tactile hair”

OR

whisker AND (sens* OR feeling OR perception)

AND

temperature OR heat OR thermal OR “temperature extremes” OR cold OR weather OR season OR seasonality OR sunlight OR climate OR growth OR moult OR molt OR shedding OR wear OR wind OR precipitation OR turbulence OR rain OR wet OR “water movement” OR hydrodynamic OR flow OR turbid OR tide OR current OR vision OR light OR dark OR sediment OR eutrophication OR turbidity OR acidification OR acid* OR pH OR health OR disease OR pathogens OR virus OR “Habitat change” OR “habitat loss” OR “habitat fragmentation” OR “land use” OR “landscape change” OR impoverished OR noise OR acoustic OR vibration OR sound OR Electric OR conductance OR EMF OR “electromagnetic sensing” OR electrosensing OR magnetic OR “heavy metals” OR pesticides OR chemicals OR pollutants OR toxins OR contaminants OR pharmaceuticals OR drugs OR “pharmaceutical contamination” OR bioaccumulation OR “endocrine disruptors” OR pollution OR food OR eutrophication OR hypoxia OR drought OR nutrients

NOT

"lignin whisker*" OR "whisker-like" OR "nanochitin whisker" OR "box-whisker" OR "box whisker" OR "box and whisker" OR chemistry OR engineering OR “material* science” OR nanowhisker* OR "nano-whisker" OR "cellulose whisker" OR "ceramic whisker" OR "crystal whisker" OR "metal whisker" OR nanowhiskers OR composite* OR “3D-print*” OR “3-D Print*” OR “3D print*” OR “artificial whisker”

Scopus Search: 2008 documents

- In: Title, abstract, keywords
- limit to: Neuroscience, Agriculture & Biological science, Medicine, Multidisciplinary, Environmental science, psychology, veterinary
- limit to: Article, conference paper, letter, data paper

(TITLE-ABS-KEY (vibriss* OR "tactile hair") OR TITLE-ABS-KEY (whisker AND (sens* OR feeling OR perception)) AND TITLE-ABS-KEY (temperature OR heat OR thermal OR "temperature extremes" OR cold OR weather OR season OR seasonality OR sunlight OR climate OR growth OR moult OR molt OR shedding OR wear OR wind OR precipitation OR turbulence OR rain OR wet OR "water movement" OR hydrodynamic OR flow OR turbid OR tide OR current OR vision OR light OR dark OR sediment OR eutrophication OR turbidity OR acidification OR acid* OR ph

Journal of Experimental Biology • Supplementary information

OR health OR disease OR pathogens OR virus OR "Habitat change" OR "habitat loss" OR "habitat fragmentation" OR "land use" OR "landscape change" OR impoverished OR noise OR acoustic OR vibration OR sound OR electric OR conductance OR emf OR "electromagnetic sensing" OR electrosensing OR magnetic OR "heavy metals" OR pesticides OR chemicals OR pollutants OR toxins OR contaminants OR pharmaceuticals OR drugs OR "pharmaceutical contamination" OR bioaccumulation OR "endocrine disruptors" OR pollution OR food OR eutrophication OR hypoxia OR drought OR nutrients) AND NOT TITLE-ABS-KEY ("lignin whisker*" OR "whisker-like" OR "nanochitin whisker" OR "box-whisker" OR "box whisker" OR "box and whisker" OR chemistry OR engineering OR "material* science" OR nanowhisker* OR "nano-whisker" OR "cellulose whisker" OR "ceramic whisker" OR "crystal whisker" OR "metal whisker" OR nanowhiskers OR composite* OR "3D-print*" OR "3-D Print*" OR "3D print*" OR "artificial whisker")) AND (LIMIT-TO (SUBJAREA , "ENGI") OR LIMIT-TO (SUBJAREA , "BIOC") OR LIMIT-TO (SUBJAREA , "PHYS") OR LIMIT-TO (SUBJAREA , "MATE") OR LIMIT-TO (SUBJAREA , "COMP") OR LIMIT-TO (SUBJAREA , "PHAR") OR LIMIT-TO (SUBJAREA , "CHEM") OR LIMIT-TO (SUBJAREA , "CENG") OR LIMIT-TO (SUBJAREA , "IMMU") OR LIMIT-TO (SUBJAREA , "MATH") OR LIMIT-TO (SUBJAREA , "ARTS") OR LIMIT-TO (SUBJAREA , "HEAL") OR LIMIT-TO (SUBJAREA , "NURS") OR LIMIT-TO (SUBJAREA , "ENER") OR LIMIT-TO (SUBJAREA , "EART") OR LIMIT-TO (SUBJAREA , "DENT") OR LIMIT-TO (SUBJAREA , "SOCI") OR LIMIT-TO (SUBJAREA , "DECI") OR LIMIT-TO (SUBJAREA , "Undefined") OR LIMIT-TO (SUBJAREA , "BUSI") OR LIMIT-TO (SUBJAREA , "ECON")) AND (LIMIT-TO (DOCTYPE , "ar") OR LIMIT-TO (DOCTYPE , "cp") OR LIMIT-TO (DOCTYPE , "le") OR LIMIT-TO (DOCTYPE , "dp"))

Pubmed search: 985 documents

- In: Title, abstract
- Limit to: books and documents, classical article, comparative study, multicenter study, observational study veterinary, overall, practice guidelines, research support (NIH, non-US gov't, US gov't), technical report
- Limit to: Other animals

(((vibriss*[Title/Abstract] OR "tactile hair"[Title/Abstract]) OR (whisker[Title/Abstract] AND (sens*[Title/Abstract] OR feeling[Title/Abstract] OR perception[Title/Abstract]))) AND (temperature[Title/Abstract] OR heat[Title/Abstract] OR thermal[Title/Abstract] OR "temperature extremes"[Title/Abstract] OR cold[Title/Abstract] OR weather[Title/Abstract] OR season[Title/Abstract] OR seasonality[Title/Abstract] OR sunlight[Title/Abstract] OR climate[Title/Abstract] OR growth[Title/Abstract] OR moult[Title/Abstract] OR molt[Title/Abstract] OR shedding[Title/Abstract] OR wear[Title/Abstract] OR wind[Title/Abstract] OR precipitation[Title/Abstract] OR turbulence[Title/Abstract] OR rain[Title/Abstract] OR wet[Title/Abstract] OR "water movement"[Title/Abstract] OR hydrodynamic[Title/Abstract] OR flow[Title/Abstract] OR turbid[Title/Abstract] OR tide[Title/Abstract] OR current[Title/Abstract] OR vision[Title/Abstract] OR light[Title/Abstract] OR dark[Title/Abstract] OR sediment[Title/Abstract] OR eutrophication[Title/Abstract] OR turbidity[Title/Abstract] OR acidification[Title/Abstract] OR acid*[Title/Abstract] OR pH[Title/Abstract] OR health[Title/Abstract] OR disease[Title/Abstract] OR pathogens[Title/Abstract] OR virus[Title/Abstract] OR "Habitat change"[Title/Abstract] OR "habitat loss"[Title/Abstract] OR "habitat fragmentation"[Title/Abstract] OR "land use"[Title/Abstract] OR "landscape change"[Title/Abstract] OR impoverished[Title/Abstract] OR noise[Title/Abstract] OR

Journal of Experimental Biology • Supplementary information

acoustic[Title/Abstract] OR vibration[Title/Abstract] OR sound[Title/Abstract] OR Electric[Title/Abstract] OR conductance[Title/Abstract] OR EMF[Title/Abstract] OR "electromagnetic sensing"[Title/Abstract] OR electrosensing[Title/Abstract] OR magnetic[Title/Abstract] OR "heavy metals"[Title/Abstract] OR pesticides[Title/Abstract] OR chemicals[Title/Abstract] OR pollutants[Title/Abstract] OR toxins[Title/Abstract] OR contaminants[Title/Abstract] OR pharmaceuticals[Title/Abstract] OR drugs[Title/Abstract] OR "pharmaceutical contamination"[Title/Abstract] OR bioaccumulation[Title/Abstract] OR "endocrine disruptors"[Title/Abstract] OR pollution[Title/Abstract] OR food[Title/Abstract] OR eutrophication[Title/Abstract] OR hypoxia[Title/Abstract] OR drought[Title/Abstract] OR nutrients[Title/Abstract])) NOT ("lignin whisker*"[Title/Abstract] OR "whisker-like"[Title/Abstract] OR "nanochitin whisker"[Title/Abstract] OR "box-whisker"[Title/Abstract] OR "box whisker"[Title/Abstract] OR "box and whisker"[Title/Abstract] OR chemistry[Title/Abstract] OR engineering[Title/Abstract] OR "material* science"[Title/Abstract] OR nanowhisker*[Title/Abstract] OR "nano-whisker"[Title/Abstract] OR "cellulose whisker"[Title/Abstract] OR "ceramic whisker"[Title/Abstract] OR "crystal whisker"[Title/Abstract] OR "metal whisker"[Title/Abstract] OR nanowhiskers[Title/Abstract] OR composite*[Title/Abstract] OR "3D-print*"[Title/Abstract] OR "3-D Print*"[Title/Abstract] OR "3D print*"[Title/Abstract] OR "artificial whisker"[Title/Abstract])

Both searches downloaded as RIS files including abstracts. 2993 records entered into Rayyan.

Duplicates

- 2993 articles
- Look for duplicates – DOI and title
- 456 duplicates identified automatically using Rayyan and removed

Screening

2537 articles to screen using title and abstracts. 2367 rejected based on:

- Article Type - 41
- Not animal vibrissa - 545
- Not relevant data - 279
- Not relevant environment-related stimulus - 1502

170 articles to screen using full-text and 44 rejected, based on:

- Duplicates - 1
- Article Type - 3
- Not animal vibrissa –14
- Not relevant data –6
- Not relevant environment-related stimulus –20

30% of articles double-screened at this stage for agreement, with 98.1% agreement.

Extraction

126 articles included and coded as per table 1 in main text document, and main findings summarised in Table S1.

Journal of Experimental Biology • Supplementary information

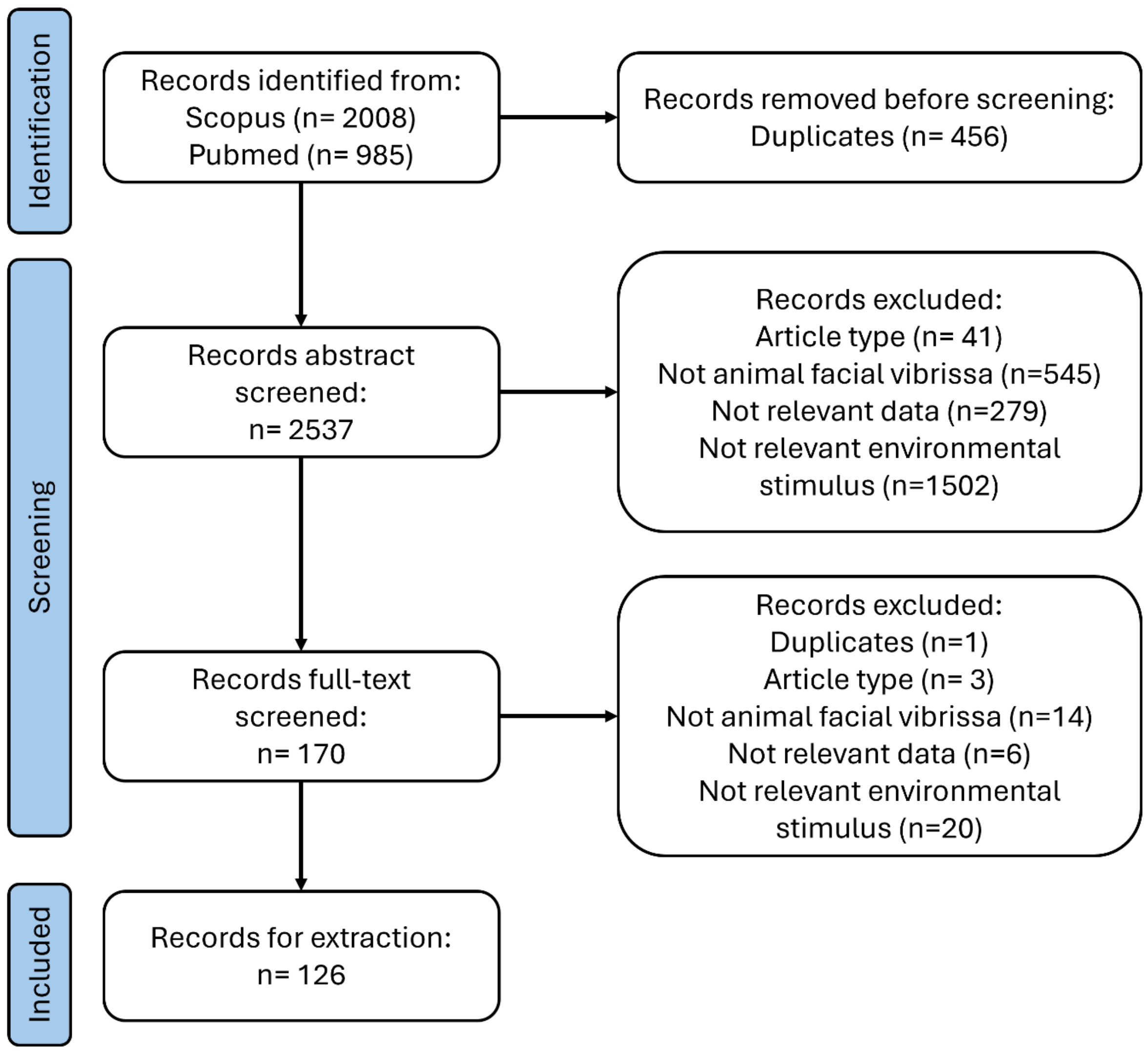

Fig. S1. PRISMA diagram showing breakdown of paper screening and shortlisting

Journal of Experimental Biology • Supplementary information

Table S1. Summary of 126 included papers featuring in the systematic review. Species identified and environmental factors coded as: HL: Habitat and land change; T: Temperature; FW: Food and Water; W: Weather; C: Chemical; A: Acoustic; N: Nutrients; E: Electrical; L: Light; IS: Invasive Species; HD: Health, Disease and Pathogens. Lab (laboratory) rats are bred from brown rats, and lab mice from house mice.

Authors	Species common names	Env. Factor Code	Stimulus	Main Findings
Habitat-related papers				
Devonshire IM, Dommett EJ, Grandy TH, Halliday AC, Greenfield SA.	lab rat	HL	Enrichment	Somatosensory evoked recordings in barrel cortex had higher responses in enriched animals, but no real difference in thalamocortical responses
LeMessurier AM, Laboy-Juarez KJ, McClain K, Chen S, Nguyen T, Feldman DE	lab mouse	FW	enrichment	Mice with enrichment has whisker tuning in L4 and improved whisker point representations and created boundaries in L2/3. Enrichment improved functional column topography in S2, perhaps improving touch detection in enriched animals
Arkley K, Tiktak GP, Breakell V, Prescott TJ, Grant RA.	dormouse	HL	habitat gaps, movement	Dormice whisk their whiskers to cross gaps, but when gaps are widened, they spend more time on floor travelling, which has implications for habitat fragmentation
Arkley K, Grant RA, Mitchinson B, Prescott TJ.	lab rat	HL	habitat, movement	In dark environments with high risks of collision, rats move slower with more whisker movements. In open environments that are familiar, they move faster and reduce whisker movements
Seo ML.	lab rat	HL	enrichment	The epicortical responses to whisker touch were analysed, and there was shortening of the latency of the response in the enriched animals
Blanchard RJ, Dulloog L, Markham C, Nishimura O, Nikulina Compton J, Jun A, Han C, Blanchard DC.	lab rat	HL	maze underground	Submissive individuals would guard tunnels, dominant individuals would lunge at them and withdraw once their whiskers had contacted
Ayaz A, Stäuble A, Hamada M, Wulf MA, Saleem AB, Helmchen F.	lab mouse	HL	running and environment	Locomotion enhances barrel cortex activity, with L5 reporting touch mainly, and L2/3 integrating touch and running
Eyre B, Shaw K, Sharp P, Boorman L, Lee L, Shabir O, Berwick J, Howarth C.	lab mouse	HL	Locomotion and environment	If locomotion occurs before or during a whisker stimulus the amplitude of the haemodynamic response was altered
Air and water flow papers				
Yu YSW, Graff MM, Hartmann MJZ.	lab rat	W, HL	flow regime	Whiskers bend in the direction of airflow and their bending magnitude increases with airflow speed. Identifies them as multimodal sensors
Yu YS, Graff MM, Bresee CS, Man YB, Hartmann MJ.	lab rat	W, HL	airflow	Vibrissal removal affected performance on an airflow localisation task. Whiskers help with anemotaxis
Yu YSW, Bush NE, Hartmann MJZ.	lab rat	W, HL	airflow	Whisker vibration responses depend on airspeed and shape of the whisker.

Journal of Experimental Biology • Supplementary information

				Vibrations transfer from parallel to perpendicular as airflow speed increases
Mugnaini M, Mehrotra D, Davoine F, Sharma V, Mendes AR, Gerhardt B, Concha-Miranda M, Brecht M, Clemens AM.	lab rat	W, HL	wind sensing	Supraorbital, caudal and dorsal whiskers can sense wind, and rats turn towards the wind. When these whiskers are removed they turn towards the wind less.
Ollerenshaw DR, Bari BA, Millard DC, Orr LE, Wang Q, Stanley GB.	lab rat	W, HL	air puffs	Rats could detect small air puffs, but were worse at it when they moved their whiskers a lot, suggesting that self-motion might provide noise to the system
Kim HJ, Yoon HS.	harbour seal	T, W, HL	water movements, temperature	The undulations of seal whiskers impact both thermal conduction and forces felt from water movements
Hanke W, Meyer S, Bleckmann H, Dehnhardt G.	Australian water rat	W, HL	diet, flow	The animals did not respond to surface waves, but did respond to hydrodynamic stimuli and could detect them at very small amplitudes (1-9.5 mm/s)
Wang S, Liu YZ.	harbour seal	W, HL	flow	At 0-30 angles of attack the whiskers are very stable, at 60-90 there is some instability, and the whiskers start to oscillate
Krüger Y, Hanke W, Miersch L, Dehnhardt G.	harbour seal	W, HL	hydrodynamics, diet	Harbour seals can detect and analyse direction of single vortex rings
Wieskotten S, Dehnhardt G, Mauck B, Miersch L, Hanke W.	harbour seal	W, HL	hydrodynamics	A blindfolded seal could detect the direction of paddles even when the trail was 35 s old
Gaspard JC, Bauer GB, Reep RL, Dziuk K, Read L, Mann DA.	manatee	W, HL	water flow	Facial vibrissae could be used to detect water movements below the hearing of manatees, at displacements of 1um for 15-150 Hz and 1nm at 150 Hz
Wieskotten S, Mauck B, Miersch L, Dehnhardt G, Hanke W.	harbour seal	W, HL	water flow	Seals used multiple water parameters, velocities, gradients and spaces of wakes to detect size and shape differences of hydrodynamic signatures of shapes
Gläser N, Wieskotten S, Otter C, Dehnhardt G, Hanke W.	California sea lion	W, HL	hydrodynamics	California sea lions can follow hydrodynamic trails left by submarines, but with less performance than harbour seals
Reep RL, Gaspard JC, Sarko D, Rice FL, Mann DA, Bauer GB.	manatee	W, HL	hydrodynamics	Manatee body whiskers and facial whiskers are used for hydrodynamic sensing, but only facial whiskers are used for touch
Hanke W, Witte M, Miersch L, Brede M, Oeffnet J, Michael M, Hanke F, Leder A, Dehnhardt G.	harbour seal	W, HL	hydrodynamics	Harbour seal whisker undulations reduce vortex-induced vibrations almost a whole order of magnitude more than non-undulating sea lion whiskers
Murphy CT, Reichmuth C, Eberhardt WC, Calhoun BH, Mann DA.	harbour seal	W, HL	hydrodynamic, vibrations	Seal whiskers vibrate with airflow 100-300 Hz with a dynamic response, and overall match well with observations from texture tasks
Murphy CT, Marx M, Martin WN, Jiang H, Lapseritis JM, French AN, Simmons NB, Moore MJ.	North Atlantic right whale	W, HL, FW	water movements, diet	Hairs are on the leading edge of the head and arranged in an ordered way. The size of the hair is similar to plankton; therefore, they probably sense water movements from plankton during foraging
Niesterok B, Dehnhardt G, Hanke W.	harbour seal	W, HL, FW	water movements, diet	Were able to detect hydrodynamic stimuli (like flatfish breathing) in open water while

Journal of Experimental Biology • Supplementary information

				moving. Noise of water and self probably impact a little. Were more successful at the task when swam slower with the more challenging, smaller stimuli
Wieskotten S, Dehnhardt G, Mauck B, Miersch L, Hanke W.	harbour seal	W, HL, FW	hydrodynamics	Many fish swim with a burst and glide stage and a submarine was used to simulate this. The glide phase impacted the ability of the seal to track the trail when trails were >15s old. Smaller movements are likely to be impacted more
Adachi T, Naito Y, Robinson PW, Costa DP, Hückstädt LA, Holser RR, Iwasaki W, Takahashi A	elephant seal	W, HL, FW	hydrodynamics	Elephant seals can detect hydrodynamic signals from prey in the field, and can rhythmically move their whiskers too
Schulte-Pelkum N, Wieskotten S, Hanke W, Dehnhardt G, Mauck B.	harbour seal	W, HL	hydrodynamics	Harbour seals can track conspecifics from water movements, usually crossing the trail in an undulatory fashion
Zhao H, Zhang Z, Chen W, Zhao Y, Sefah EY, Ji C, Yuan D.	harbour seal	W, HL	upstream wake	Whiskers had high sensitivity to changes in pitching amplitude and motion frequency of upstream wakes, less affected by heaving amplitude
Wei Y, Ji C, Yuan D, Song L, Xu, D.	harbour seal	W, HL	flow induced vibration	Whiskers suppress vibrations with flow angles of 0, but at >30 the whisker loses its suppression ability, resulting in large-amplitude vibration responses (similar to a cylinder)
Murphy CT, Reichmuth C, Mann D.	harbour seal	W, HL	water movement. Flow vibrations	Seal whiskers were sensitive to a vibrating plate from 20-250 Hz, wider than the usual range of water vibrations
		Chemical papers		
Kang S, Hayashi Y, Bruyns-Haylett, Baker DH, Boura M, Wang X, Karatzas K-A, Serra I, Bithell A, Williams C, Field DT, Zheng Y.	lab rat	C	Vitamin B12	Dietary supplements of vitamin B12 caused increases (25%) on Local field potentials in cortex following whisker stimulation. Spontaneous neural activity was not affected. Maybe B12 rats have enhanced sensitivity to sensory stimulation
Sainsbury KA, Shore RF, Schofield H, Croose E, Pereira MG, Sleep D, Kitchener AC, Hantke G, McDonald RA.	pole cat	C	anticoagulant rodenticides	Most (54/68) animals had residues of second-generation anticoagulant rodenticides in their whiskers, higher in arable areas in the east of the UK
McCallister MM, Maguire M, Ramesh A, Aimin Q, Liu S, Khoshbouei H, Aschner M, Ebner FF, Hood DB.	lab rat	C	Benzo[a]pyrene (BaP), hydrocarbon	BaP exposed offspring had 70% reduced neural activity compared to controls when a whisker was deflected
Banerjee U, Burks TF, Feldberg W, Goodrich CA.	rabbit	C	5-hydroxytryptophan (5-HTP) and 5-hydroxytryptamine (5-HT)	Injections caused rapid movements of the whiskers and nodding and sideways movements of the head
White CM, Giarikos DG, Hirons AC.	Northern fur seal	C	organic pollutants	Organochlorine contaminants and polybrominated diphenyl ether contaminants were present in the vibrissae, what this means for the health of the marine mammal is not yet known. Perhaps indicates that POP contaminants can collect in inert

				tissues where they cannot cause toxic effects
García-Fernández RA, Pérez-Martínez C, Escudero-Diez A, García-Iglesias MJ	lab mouse	C	Retinoic Acid	Pre-natal exposure to all-trans retinoic acid had no difference in the distribution of keratin in mice, compared to controls
Starr MA, Page ME, Waterhouse BD.	lab rat	C	MDMA	Short-term low dose MDMA increased firing and reduced whisker-simulated activity in rats. Tactile signals are distorted
Klein BG, McCain WC, Ehrich M.	lab rat	C	Carbamate exposure	No difference of exposure (at relatively low levels) on number of myelinated axons and the areas of these axons and myelin sheaths
Khasabov SG, Truong H, Rogness VM, Alloway KD, Simone DA, Giesler GJ.	lab rat	C, T	itch and pain chemicals - chloroquine, serotonin, alanine, histamine, capsaicin, and mustard oil	Histamine activated the biggest numbers of neurons, especially around the deep laminae of the medial boarder of the vibrissal barrel fields, and the dysgranular zone. Itch and pain might be coded in the vibrissal areas
Tournissac M, Chaigneau E, Pfister S, Aydin AK, Goulam Houssen Y, O'herron P, Filosa J, Collot M, Joutel A, Charpak S.	lab mouse	C	CO2	CO_2 does not affect neurovascular coupling caused by vibrissal stimulation
Papp A, Pecze L, Szabó A, Vezér T.	lab rat	C	heavy metals, lead, mercury, manganese	Metals all caused an increase in evoked potential amplitude in the barrel field following whisker stimulation. Hg has the strongest effect, followed by Mn
Pecze L, Papp A, Nagymajtényi L, Dési I.	lab rat	C	lead, mercury, manganese	Metals all caused an increase in amplitude in the barrel field following whisker stimulation. Hg has the strongest effect, followed by Mn
Kooyomjian C, Giarikos D, Adkesson M, Hirons AC.	Peruvian fur seal, South American sea lion	C	trace element metals	Vibrissae contained trace elements, at levels 2-20 higher than the serum
Roy M, Jacque N, Novicoff W, Li E, Negash R, Evans SJM.	cat	C	Molnupiravir antiviral	Molnupiravir can be used to treat feline infectious peritonitis (and COVID-19) but can cause broken whiskers at doses higher than 23mg.kg twice daily
Zhang Y, Wang Y, Chen Z, Cheng S, Ding C, Zhang J, Peng T, Chen W, Zhang D, Tan Y, Wang X, Dong R, Jiang M, Hua Q.	lab mouse	C	tyrosine kinase inhibitors	Tyrosine kinase inhibitors led to whisker irregular growth, thickness and curvature
Drouin C, Page M, Waterhouse B.	lab rat	C	Methylphenidate	Both doses of MPH supressed responses in primary somatosensory cortex following stimulation of the whisker pad
Renard A, Harrell ER, Bathellier B.	lab mouse	C	amyl acetate and ethyl butyrate (banana and pineapple)	The presence of odours altered whisker positions and movements
Pérez AC, Demers M, Fassihi A, Moore JD, Kleinfeld D, Deschênes M.	lab rat	C	ammonia delivery	Odour delivery corresponds to a grimace and vibrissal protraction

Journal of Experimental Biology • Supplementary information

Parabucki A, Lampl I.	lab rat	C	smell, olfactory cortex	Whisker stimuli are detected in local field potentials in olfactory bulb, highly correlated to barrel cortex activation
Vanexan RJ, Hardy MH.	lab mouse	C	vitamin a	Vitamin penetrated both the epidermis and hair follicles
Jang W-J, Son T, Song S-H, Ryu IS, Lee S, Jeong C-H.	lab rat	C	Drug, Methamphetamine	Following methamphetamine self-administration, differentially expressed genes from the vibrissal follicles were associated with Parkinson's, Huntington's and Alzheimer's disease, as well as endocannabinoid signalling and the synaptic vesicle pathway
Magalhães A, Tavares MA, de Sousa L.	lab rat	C	cocaine	Young rats administered with cocaine did not show a difference in vibrissal cleaning responses compared to controls
Rutter JJ, Baumann MH, Waterhouse BD.	lab rat	C	cocaine	VPM thalamus neurons recorded in response to whisker stimulation. Cocaine increased response magnitude, but the response time of peripheral thalamic neurons were reduced
Bekavac I, Waterhouse BD.	lab rat	C	cocaine	Cocaine effected somatosensory responses following whisker stimulation, both dose and whisker identify affected the responses
Devonshire IM, Mayhew JE, Overton PG.	lab rat	C	cocaine	Increased responses to whisker touch in the primary somatosensory cortex, especially in the upper layers
Rutter JJ, Devilbiss DM, Waterhouse BD.	lab rat	C	cocaine	Cocaine enhanced responses, especially to small magnitude deflections, suggesting that cocaine may enhance detection but impede discrimination
		Sound papers		
Lohse M, Dahmen JC, Bajo VM, King AJ.	lab mouse	A	Sound and touch	Stimulating the vibrissae supressed sound-evoked activity in primary auditory cortex, which is implemented through mid-brain and thalamic routes.
Käkelä R, Hyvärinen H.	Saimaa ringed seal	A	diet, sound	Fatty acid composition around the follicle is different to that of blubber, with twice as much hexadecenoic acid and tetradecenoic acid. This is probably not affected by diet, but might be an important part of vibrissal functioning, perhaps even to translate sound signals to improve sound sensing
Clayton KK, Stecyk KS, Guo AA, Chambers AR, Chen K, Hancock KE, Polley DB.	lab mouse	A	broadband sounds	Facial motion (next to vibrissae array) was 30dB sensitive to sound than the acoustic startle reflex
Liu X, Chen G, Zang B, Azarpeyvand M.	harbour seal	A	far-field noise	Undulations reduce noise over a wide range of Strouhal numbers (0.1-2)
Rezaei Z, Jafari Z, Afrashteh N, Torabi R, Singh S, Kolb BE, Davidsen J, Mohajerani MH.	lab mouse	A	auditory stress (3000Hz of 90 dB for 24 hours)	Sound receiving auditory stress had reduced amplitude responses in their cortex to whisker stimulation.
Shatz LF, Christensen CW.	lab rat	A	sound	Sound from a stereo speaker moved the whisker with a similar amount to cricket cercel hairs or inner ear hair bundles
Di S, Brett B, Barth DS.	lab rat	A	sound, multimodal	Primary auditory and primary somatosensory areas respond to sound and

				touch, respectively, but a small poly-sensory area exists between them and receive thalamocortical projections
So EC, Chen YH, Huang CY, Chen JY, Huang BM, Poon PW.	lab rat	A	sound	Rat pups exposed to a pure tone (4kHz, 64dB, 8hr/day) accelerated the appearance of all reflexes, including vibrissal placement
Zhang M, Kwon SE, Ben-Johny M, O'Connor DH, Issa JB.	lab mouse	A	Sound and vibration	Sound mainly decreases the response of primary and secondary somatosensory areas in response to vibrations. A small population of S2 neurons only respond to sound
Webster M, Webster DB.	kangaroo rat	A	multimodal	Removing vibrissae on its own did not effect performance in a maze task, but with vision removal it did, and with deafening and blinding also
Zhu JY, Yuan YY, Hu ZW, Yang ZG, Xu JQ.	harbour seal	A	wind sound	Seal whisker shapes reduce noise by 11-13 dB, showing that they can reduce aerodynamic noise, compared to smooth cylinders
Chen G, Liu X, Zang B, Azarpeyvand M.	harbour seal	A	wind sound	Aeolian (wind) tone is supressed by undulating seal whisker, compared to other shapes, by ~10dB. The 3D shape prevents vortex shedding.
McKnight JC, Ruesch A, Bennett K	harbour seal	A, HL, N	light, sounds, touch	None of the tactile regions of the brain were associated with visual or auditory cues; nor were other brain areas associated with tactile cues, suggesting localised responses to each sense
Electric Papers				
Anselmo CWSF, Santos AAA, Freire CMA, Ferreira LMP, Cabral Filho JE, Catanho MTJA, do Carmo Medeiros M.	lab rat	E, FW	diet, electromagnetic fields	The offspring of pregnant rats exposed to a basic diet had delayed vibrissal placement responses, but EMFs had no effect on vibrissal placement responses
Weigel RJ, Lundstrom DL.	lab rat	E	electric fields and humidity	Electric fields cause movement of the vibrissae, which disappear at humidity of >39%
Hüttner T, von Fersen L, Miersch L, Czech NU, Dehnhardt G.	bottlenose dolphin	E	weak electric fields, water movements	Four bottlenose dolphins responded to water movements and weak electric fields 1.5 mV/cm. Three dolphins continued to respond to the electric fields at 0.5 mV/cm
Hüttner T, von Fersen L, Miersch L, Dehnhardt G.	bottlenose dolphin	E	DC and AC electrical fields	Two dolphins can detect DC currents (2.4 and 5.5 μV/cm) and AC currents (1, 5 and 25 Hz), to a similar detection threshold as Guiana dolphins and platypus
Czech-Damal NU, Liebschner A, Miersch L, Klauer G, Hanke FD, Marshall C, Dehnhardt G, Hanke W.	Guiana dolphin	E	electrosensing	Guiana dolphins could detect weak electric fields of 4.6 uV/cm
Orlacchio R, Percherancier Y, Poulletier De Gannes F, Hurtier A, Lagroye I, Leveque P, Arnaud-Cormos D.	lab mouse	E	radiofrequency	Brain activation was observed using functional ultrasound during whisker stroking. The radiofrequency did not affect the brain signals

Othman H, Ammari M, Rtibi K, Bensaid N, Sakly M, Abdelmelek H.	lab rat	E	Radiofrequency from conventional wifi devices	Sensorimotor impairments observed in all tests but not vibrissal placing response
Light and vision papers				
Ramamurthy DL, Krubitzer LA.	opossum	HL, N	vision multimodal	Overall tactile experience is similar between blind and sighted animals. Early blind animals have a reduction in a magnitude of responses in S1 to whisker stimulation. Single whisker stimulation reduced, but the ability to discriminate between whiskers enhanced, due to a sharpening of receptive fields
Abe K, Yawo H.	lab rat	HL, N	vision, cross-modal	When rats are deprived of vision P26-30 the sensitivity of the whiskers increased, but this was not affected by later visual deprivation (P58-66)
Lee CC, Diamond ME, Arabzadeh E.	lab rat	HL, N	vision, cross-modal	Detection performance of whiskers increased when there were more whisker-based trials than visual, similarly neuronal activity in S1 responded to more whisker signals in a more whisker-based task, revealing enhanced responses to vibration. Shows prioritisation of sensory signals
Sieben K, Röder B, Hanganu-Opatz IL.	lab rat	HL, N, L	cross-modal	Oscillating visual and touch signals together increases signals in somatosensory cortex. Some connectivity between S1 and V1
Allen AE, Procyk CA, Brown TM, Lucas RJ.	lab mouse	HL, N	vision, movement	Neurons in the sensory thalamus (VPM) get excited by directional visual motion, such as head rotations and forward movement. Responses to whisker deflections is highest when simulating moving forwards
Arruda BP, Cruz-Ochoa NA, Serra FT, Xavier GF, Nogueira MI, Takada SH.	lab rat	HL, N	environmental light cycles, anoxia, melatonin	Anoxia delayed vibrissal placement across a gap, but this is reversed by melatonin
Plant and funghi papers				
Mambou HMAY, Pale S, Bopda OSM, Jugha VT, Musa NSO, Ojongnkpot TA, Wanyu BY, Bila RB, Herqash RN, Shahat AA, Taiwe GS.	lab mouse	T, IS	*Mimosa pudica*	Reduced vibrissal hyperactivity twitching following epileptic seizure
Wang C, Zang K, Tang Z, Yang T, Ye X, Dang Y.	lab mouse	T, IS	Hordenine plant defence chemical	Significantly increased hair shaft growth in vibrissal follicles
Kang J-I, Kim E-J, Kim M-K, Jeon Y-J, Kang S-M, Koh Y-S, Yoo E-S, Kang H-K.	lab mouse	T	species movements, invasive species, algae *Ishige sinicola*	Extract of the algae increased hair shaft length and the number of dermal papilla cells.
Kang J-I, Moon J, Kim E-J, Lee Y-K, Koh Y-S, Yoo E-S, Kang H-K, Yim D.	lab rat	T, IS	invasive species, development, wheat bran, n-hexane	The vibrissal shaft lengths significantly increased in length when treated with n-hexane from wheat bran
Sun YN, Cui L, Li W, Yan XT, Yang SY, Kang JI, Kang HK, Kim YH.	lab rat	T, IS	invasive species, development, *Polygonum multiflorum* roots	The vibrissal shaft lengths significantly increased in length when treated with *polygonum multiflorum* roots

Journal of Experimental Biology • Supplementary information

Rho S-S, Park S-J, Hwang S-L, Lee M-H, Chang DK, Lee I-H, Chang S-Y, Rang M-J.	lab mouse	T	plant, *Asiasari radix*	Extract of *Asiasari radix* increased protein synthesis in the vibrissal follicle
Matsuda H, Yamazaki M, Asanuma Y, Kubo M.	lab mouse	T	*Ginseng radix*	Hair shaft growth seen in vibrissal follicles treated with red ginseng roots
Sakaguchi I, Ishimoto H, Matsuo M, Ikeda N, Minamino M, Kato Y.	lab mouse	T	*Illicium anisatum*	Water soluble extracts of the leaves, fruits and roots causes hair follicle and shaft growth, but acetate and hexane soluble extracts inhibited hair and follicle growth
Towalari K, Kondo R, Sakai K.	lab rat	T	fungus YL161, *Agaricus blazei*, and the bark of *Camptotheca cuminata*	Increased dermal papillae cell growth compared to pentade canoic acid
Temperature papers				
Petrov EA, Kupchinsky AB, Fialkov VA, Badardinov AA.	Baikal seal	T	seasons	Warm winters and early springs cause ice melt and the seal does not finish moulting on ice, and hauls our on shore. Likely to affect foraging
Queiroz JPAFD, Souza JBF, Oliveira VRDM, Costa LLDM, Oliveira MFD.	Spix cavy	T	high temperatures	Vibrissae regions act as thermal windows for radiative heat reduction.
Erdsack N, Dehnhardt G, Witt M, Wree A, Siebert U, Hanke W.	harbour seal	T	temperature	High hair densities around vibrissal follicles to reduce heat loss
Mauck B, Eysel U, Dehnhardt G.	harbour seal and Guiana dolphin	T	temperature	Both species have high surface temperatures at the moth od the follicle, and likely has an independent blood supply. Likely to be a thermoregulatory system
Dehnhardt G, Mauck B, Hyvärinen H.	harbour seal	T	temperature	Seal has same discrimination thresholds doing a texture task at 1.2 C and 22 C, the follicles are heated more than surrounding skin, and likely maintain sensitivity of mechanoreceptors
Britton SW.	cat	T	temperature	At temperature 19-20 degrees a vibrissal reflex is observed
Dickenson AH, Hellon RF, Taylor DC.	lab rat, rabbit	T	temperature	Both rats and rabbits had temperature-sensitive neurones (many cold and some warm) around the nose, whisker pad and mouth. The many cold neurons are mapped topographically in the caudal trigeminal nucleus
Yoon HS, Nam SH, Kim MI.	harbour seal	T	temperature	Undulations help to increase heat transfer from the whisker into the water
Daily and seasonal papers				
Jasinska M, Grzegorczyk A, Woznicka O, Jasek E, Kossut M, Barbacka-Surowiak G, Litwin JA, Pyza E.	lab mouse	T,L	light, circadian rhythms	In mice in usual conditions, the number of excitatory synapses on the dendritic spines of neurons in barrel cortex increases in the light, rest phase of the day, and the number of inhibitory synapses are higher in the dark, active part of the night. In animals in constant darkness, there is only lots of inhibitory synapses
Jasinska M, Grzegorczyk A, Jasek E, Litwin JA, Kossut M,	lab mouse	T,L	daily rhythm	Barrel cortex in the dark, active part of the night had more total and more excitatory synapses on the dendritic spines in barrel

Journal of Experimental Biology • Supplementary information

Barbacka-Surowiak G, Pyza E.				cortex neurons. In the light, there is more inhibitory synapses
Buhr ED, Vemaraju S, Diaz N, Lang RA, Van Gelder RN.	lab mouse	T,L	seasonal, light, melanocyte precursor cells	Vibrissal skin entrains to a light-dark cycle using light
		Diet papers		
Schwarz JF, DeRango EJ, Zenth F, Kalberer S, Hoffman JI, Mews S, Piedrahita P, Trillmich F, Paez-Rosas D, Thiboult A, Krüger O.	Galapagos sea lion	T, FW	Environment, foraging	Benthically foraging individuals dive into the substrate and prey on cusk eels, their whiskers wear, and they have much shorter whiskers. Pelagic foragers remain relatively horizontal in the water and have much longer whiskers
Elliott Smith EA, Moss ML, Wellman HP, Gill VA, Monson DH, Newsome SD.	sea otter	FW	diet	Diet signatures found in vibrissae that can be tracked over time to identify changing diet shifts from bivalves to a greater recent reliance on kelp forests, using chemical analysis
Tucker L, Russell ML, Hieb EE, Carmichael RH, Cloyed CS.	manatee	FW	diet, species, temperature	Detect diet signatures in time, along the vibrissal shaft. Can also identify cold stress using isotope analysis
Walters A, Hindell M, Goebel ME, Bester MN, Trathan PN, Oosthuizen WC, Lea M-A.	Antarctic fur harbour seal	FW	diet, moving species	Isotopes can be studied to infer diet and types of waters where the seals have been foraging.
Goetz KT, Burns JM, Hückstädt LA, Shero MR, Costa DP.	Weddell seal	FW	diet isotope analysis	Analysed N and C isotopes in whiskers for diet and identified dusky rock cod and silverfish to be important in the diet, although these changed with time and individual.
Giriko CT, Andreoli CA, Mennitti LV, Hosoume LF, Souto TDS, Silva AVD, Mendes-da-Silva C.	lab rat	FW	diet, high-fat diet	Offspring of mothers fed a high-fat diet had delayed vibrissal placing responses, by over a week
Hindell MA, Lydersen C, Hop H, Kovacs KM.	bearded seal	FW	diet isotope analysis	Whiskers of newborn pups used for isotope diet analysis revealing that seals fed further offshore when ice cover is large, and moved to fjords when ice cover is less, indicated by the prey types identified from the analysis
Kupetsky-Rincon EA, Li Q, Uitto J.	lab mouse	FW	diet magnesium	Magnesium enriched diet prevents mineralisation of the vibrissae capsule, which is a marker of *psuedoxanthoma elasticum*
Berman SL.	Plains viscacha rat	FW	diet	In-growing vibrissal hairs are positioned behind the incisors and vibrate to strip epidermis from leaves during feeding, linked to extrinsic muscles
Page HM, Schamel J, Emery KA, Schooler NK, Dugan JE, Guglielmino A, Schroeder DM, Palmstrom L, Hubbard DM, Miller RJ.	island fox	FW	diet, isotope	Diet analysis can be conducted in the whiskers of island fox,
Lerner JE, Ono K, Hernandez KM, Runstadler JA, Puryear WB, Polito MJ.	grey seal	FW	diet, isotope	Isotope analysis can be used to study diet in grey seals

Journal of Experimental Biology • Supplementary information

Rosas-Hernández MP, Hernández-Camacho CJ, González-Rodríguez E, Aurioles-Gamboa D.	California sea lion	FW	diet, isotope	Whisker growth rates vary by sex, age, individual and species. The isotope N and C values can be used for diet analysis
Calvo-Mac C, Ugarte-Barriga A, Canales-Cerro C, Klarian SA, Cárcamo C, Vargas-Pérez J, Medina-Vogel G.	American mink, cat	FW	diet, isotope	Can look at dietary isotope analyses in these species
Sepúlveda M, Newsome SD, Pavez G, Oliva D, Costa DP, Hückstädt LA.	South American sea lion	FW	diet, isotope	Isotope analysis, used in collaboration with GPS tags, can inform effect of predating on salmon farms
Pyatskowit JW, Prohaska JR.	lab rat	FW, C	copper deficiency	Copper deficient rats had impaired vibrissal-guided foot placements
Amichai E, Boerma DB, Page RA, Swartz SM, Ter Hofstede HM.	nectivorous bats	FW, HL	nectivorous bats guiding hovering flight for feeding	Nectar feeding bats have longer whiskers than non-nectivorous bats. Bat flight trajectory during hovering was impacted when whiskers were removed
Tyrrell LP, Newsome SD, Fogel ML, Viens M, Bowden R, Murray MJ.	sea otter	FW, T	diet, growth	Vibrissae growth was linear, and they could be used for isotope analysis
Edwards MR, Cárdenas-Alayza S, Adkesson MJ, Daniels-Abdulahad M, Hirons AC.	Peruvian fur harbour seal	FW, T	diet, temp	Chemical isotope analysis of the seal whiskers reveals carbon and nitrogen signatures that link with diet
Health and disease papers				
Kimura T.	house musk shrew	HD	hair follicle tumours	The histological structure of the trichoblastomas were different in the tactile hair skin, than those in humans, cats, dogs and other wildlife and face, head and neck skin.
Boukhvalova MS, Mortensen E, Mbaye A, Lopez D, Kastrukoff L, Blanco JCG.	cotton rat	HD	Herpes simplex virus 1	Herpes virus via a lip scratch caused defective whisker touch - less responsive to whisker touch
Shimatsu T, Shinozaki H, Kimitsuki K, Shiwa N, Manalo DL, Perez RC, Dilig JE, Yamada K, Boonsriroj H, Inoue S, Park C-H.	dog	HD	rabies virus	In 60 rabid dogs, the virus antigen was detected in the outer root sheath of the ring sinus of the whisker follicles
Pletnikov MV, Rubin SA, Carbone KM, Moran TH, Schwartz G.J.	lab rat	HD	Neonatal Borna disease virus	Did not observe a difference in vibrissal placement response compared to controls

Journal of Experimental Biology • Supplementary information
